# Supplementary material for: Identification of Two Novel R2R3-MYB Transcription factors, PsMYB114L and PsMYB12L, Related to Anthocyanin Biosynthesis in Paeonia suffruticosa
Source: Int J Mol Sci. 2019 Feb 28;20(5):1055. doi: 10.3390/ijms20051055 (PMC6429501; doi:10.3390/ijms20051055)
Supplement: Supplementary file 1 [file ijms-20-01055-s001.pdf]

International Journal of
Molecular Sciences

MDPI

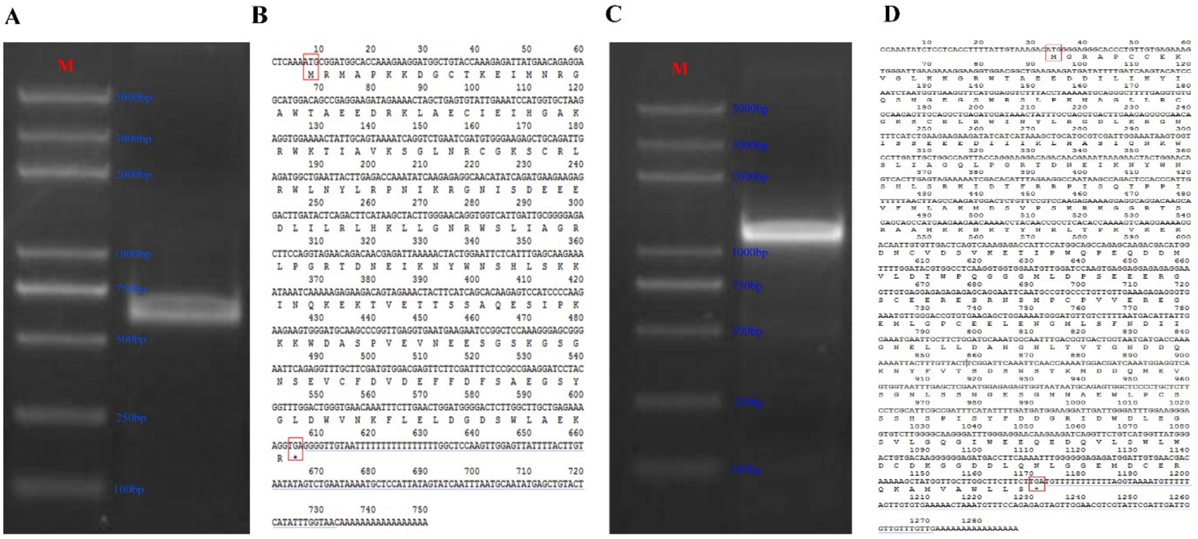

Figure S1. Full-length cDNA amplification of the *PsMYB114L* and *PsMYB12L* genes. **(A)** Results of PCR detection of the *PsMYB114L* gene. **(B)** The full-length cDNA sequence of the *PsMYB114L* gene. **(C)** Results of PCR detection of the *PsMYB12L* gene. **(D)** The full-length cDNA sequence of the *PsMYB12L* gene. ATG (start codon) and TAA (termination codon) are shown in the red box. M, Marker. The sequence of the 3 UTR is underlined in blue.

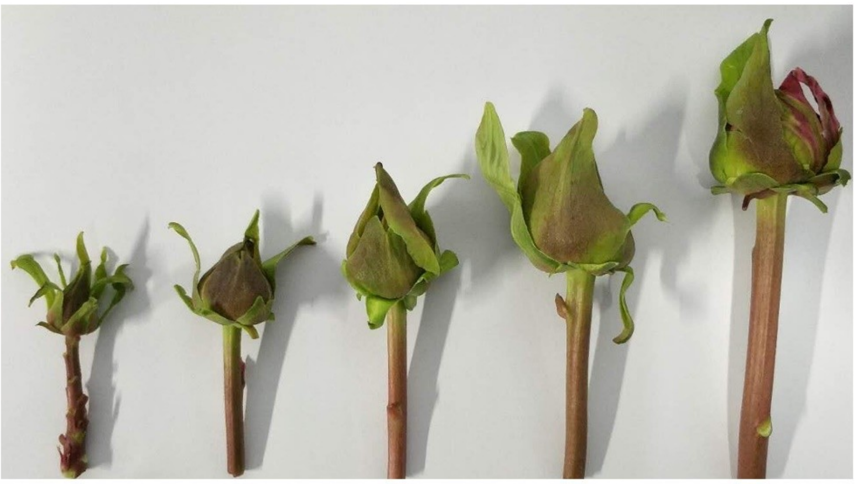

Figure S2. Flowers of *P. suffruticosa* 'Shima Nishiki' at five developmental stages. S1, flower bud emerging stage; S2, small bell-like flower-bud stage; S3, large bell-like flower-bud stage; S4, bell-like flower-bud extending stage; S5, color exposing stage.

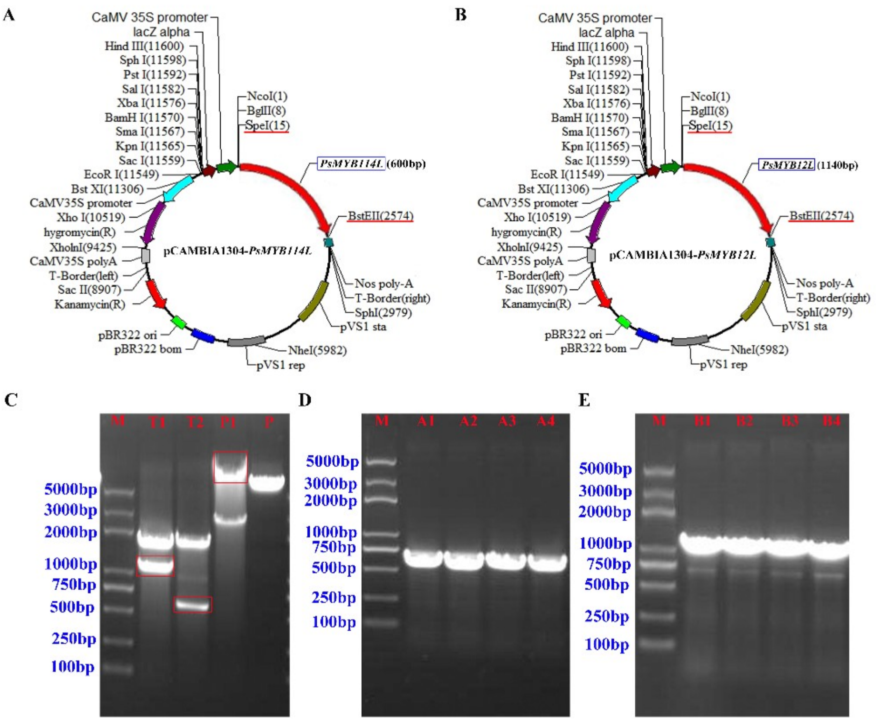

Figure S3. Construction of the recombinant expression vectors of pCAMBIA1304-*PsMYB114L* and pCAMBIA1304-*PsMYB12L*. Maps of the recombinant vectors pCAMBIA1304-*PsMYB114L* **(A)** and pCAMBIA1304-*PsMYB12L* **(B)**. The restriction sites (*Spe* I and *BstE* II) are underlined in red. **(C)** Double digestion of the vector and target gene. P, The empty vector of pCAMBIA1304; P1, Double digestion of the pCAMBIA1304 empty vector; T1, Double digestion of the pTOPO-Blunt Simple vector carrying the *PsMYB12L* gene; T2, Double digestion of the pTOPO-Blunt Simple vector carrying the *PsMYB114L* gene. The fragments used to construct the recombinant vector are shown in the red box. **(D)** PCR verification of the recombinant pCAMBIA1304-*PsMYB114L* vector. A1, A2, A3, and A4 are the four replicates used for PCR amplification. **(E)** PCR verification of the recombinant pCAMBIA1304-*PsMYB12L* vector. B1, B2, B3, and B4 are the four replicates used for PCR amplification.

Table S1. Primers used in this study.

Primer name	Sequence (5'-3')	Description
PsMYB114L-1-F	GAGGCAACATATCAGATGAAGAAGAGG	Primers used for 3′RACE amplification
PsMYB114L-2-F	CTACTTCATCAGCACAAGAGTCCATCC	
PsMYB12L-1-F	AATGGCAATTTGACGGTGACTGGT	
PsMYB12L-2-F	TGATGGAAGGATTGATTGGGATTTG	
B26	GACTCGAGTCGACATCGATTTTTTTTTTTTTTTTT	
PsMYB114L-F1	ATGCGGATGGCACCAAAGAAG	Primers used for Full-length cDNA amplification
PsMYB114L-R1	TCACCTTTTCTCAGCAAGCCAAG	
PsMYB12L-F1	ATGGGGAGGGCACCCTGTTGT	
PsMYB12L-R1	TCAAGAAAGAAGCCAAGCAACCAT	

PsMYB114L-GFPF	GGTACCATGCGGATGGCACCAAAGAA	Primers used for subcellular localization vector construction
PsMYB114L-GFPR	TCTAGACCTTTTCTCAGCAAGCCAAG	
PsMYB12L-GFPF	GGTACCATGGGGAGGGCACCCTGTTGT	
PsMYB12L-GFPR	TCTAGAAGAAAGAAGCCAAGCAACCA	
PsMYB114L-F2	ACTAGTATGCGGATGGCACCAAAGAAG	Primers used for overexpression vector construction
PsMYB114L-R2	GGTCACCTCACCTTTTCTCAGCAAGCCAAG	
PsMYB12L-F2	ACTAGTATGGGGAGGGCACCCTGTTGT	
PsMYB12L-R2	GGTCACCTCAAGAAAGAAGCCAAGCAACCAT	
1304Ve-F	GGACTCTTGACCATGGTAGATCTG	
PsCHS-F	AGCAGAGAACAACAAAGGGTCACG	Primers used for qRT-PCR
PsCHS-R	TCAGCACCGACAATAACCGCAG	
PsCHI-F	TCCCACCTGGTTCTTCTA	
PsCHI-R	AACTCTGCTTTGCTTCCG	
PsF3H-F	CCCAAGGTAGCCTACAACCAA	
PsF3H-R	GAAAATCCCCCAGTCTTCACA	
PsF3'H-F	AACTTGTTCACGGCAGGGACT	
PsF3'H-R	GGCTTGGGCTAGGATTTTAGG	
PsDFR-F	TTCATCGGTTCATGGCTTGTC	
PsDFR-R	AATGGGTATCCGCTTTTGGC	
PsANS-F	GCCCTCACTTTCATCCTCCACAAC	
PsANS-R	AAAACTGCCCACGAAATCCTTACCT	
PsFLS-F	GAATGAACAGCCTGCAATCA	
PsFLS-R	TCTCGACTAGCCTCCACCAT	
PsANR-F	ATGGCTGGTCCTTCACTTACT	
PsANR-R	CGAAATGGATATGGAACCTGA	
PsMYB114L-F	GGGAGCGGGAATTCAGAGGTT	
PsMYB114L-R	AGCCAAGAGTCCCCATCCAG	
PsMYB12L-F	ATGGCAGCCAGAGCAAGACG	
PsMYB12L-R	TCAACAACAGGGCACGGCAT	
PsUbiquintin-F	GACCTATACCAAGCCGAAG	
PsUbiquintin-R	CGTTCCAGCACCACAATC	
AtCHS-F	ACGGACATTTGAGGGAAGTTGG	
AtCHS-R	AGGGTGGGCTATCCAGAAGAGG	
AtCHI-F	GCGGTTCTGGAATCTATCATCG	
AtCHI-R	TCGTCCTTGTTCTTCATCATTAGC	
AtF3H-F	CTGACCCTGGAACCATTACCTTG	
AtF3H-R	CAGCATTCTTGAACCTCCCATT	
AtF3'H-F	ACACCGATGGAGACTGTTGAGAA	
AtF3'H-R	GCGTTAGCGTTCCAACCTCTTC	

AtDFR-F	GCCAAACGCCAAGACGCTA
AtDFR-R	CATTCACTGTCGGCTTTATCACTTC
AtANS-F	AGGTTAGGATTTCTTGGGCTGTG
AtANS-R	CCGTGGAGGAAACTTAGCCG
AtFLS-F	ACCGTTTGCTTTCAAGGATTACA
AtFLS-R	AAATGATCACCGATTTTTTTCCGTG
AtANR-F	TTCAAGAACTTGGCGACCTG
AtANR-R	CGGAGTTGCGACATGGAAGA
AtActin2-F	TGCTGGATTCTGGTGATGGT
AtActin2-R	AAGGTCAAGACGGAGGATGG
MdCHS-F	GGCAAGTGCTGTCGGATT
MdCHS-R	CCCAAAGAAATAACCACAAG
MdCHI-F	GCTACAAATGCGGTGATAG
MdCHI-R	CGCCTCCACTACAACCTCC
MdF3H-F	GCCGATCACCTACACCGAG
MdF3H-R	GTACAAGAAGTGGGAAGGC
MdF3'H-F	TTGCGGATGGTGTCTCTGATG
MdF3'H-R	GCCTCGTCCATGTTCAACTTCTC
MdDFR-F	GTTGAGGGAGATAGGGTTTGAG
MdDFR-R	GGTAAATGTAAAACAATAGAGAGG
MdANS-F	GGAGAAGATCATCCTTAAGCCA
MdANS-R	CTAAGATATATCATACCAACTATGCC
MdFLS-F	GGATAAGACAAGAATCTCATGGC
MdFLS-R	CACACCACTCACAACTTTACC
MdANR-F	TCAACAAAAGATACCCCCAG
MdANR-R	GATAGCTAGCTCGATACATGC
MdActin-F	ACACGGGGAGGTAGTGACAA
MdActin-R	CCTCCAATGGATCCTCGTTA
